# Cation Crosslinking-Induced Stable Copper Nanoclusters Powder as Latent Fingerprints Marker

**DOI:** 10.3390/nano11123371

**Published:** 2021-12-12

**Authors:** Yi Qiu, Zhuoqi Wen, Shiliang Mei, Jinxin Wei, Yuanyuan Chen, Zhe Hu, Zhongjie Cui, Wanlu Zhang, Fengxian Xie, Ruiqian Guo

**Affiliations:** 1 Institute for Electric Light Sources, School of Information Science and Technology, Fudan University, Shanghai 200433, China; 19210720021@fudan.edu.cn (Y.Q.); meishiliang@fudan.edu.cn (S.M.); 20210720024@fudan.edu.cn (J.W.); 19210720022@fudan.edu.cn (Y.C.); 20110720020@fudan.edu.cn (Z.H.); 19110720020@fudan.edu.cn (Z.C.); fdwlzhang@fudan.edu.cn (W.Z.); xiefengxian@fudan.edu.cn (F.X.); 2 Institute of Future Lighting, Academy for Engineering and Technology, Fudan University, Shanghai 200433, China; 19110860003@fudan.edu.cn; 3 Zhongshan-Fudan Joint Innovation Center, Zhongshan 528437, China; 4 Yiwu Research Institute, Fudan University, Chengbei Road, Yiwu 322000, China

**Keywords:** copper nanoclusters powder, cation crosslinking method, stability, latent fingerprints visualization

## Abstract

Luminescent copper nanoclusters (Cu NCs) have shown great potential in light-emitting devices (LEDs), chemical sensing, catalysis and biological fields. However, their practical use has been restricted by poor stability, and study on the stability of Cu NCs solid powder along with the mechanism is absent. In this study, stablized Cu NCs powder was first obtained by cation crosslinking method. Compared with the powder synthesized by solvent precipitation method, the stability of Cu NCs powder crosslinked by ionic inducer Ce^3+^ was enhanced around 100-fold. The storage time when the fluorescence intensity decreased to 85% (T_85_) was improved from 2 h to 216 h, which is the longest so far. The results of characterizations indicated that the aggregation structure was formed by the binding of Ce^3+^ with the capping ligands of Cu NCs, which helped in obtaining Ce-Cu NCs powder from aggregate precipitation in solution. Furthermore, this compact structure could avoid the destruction of ambient moisture resulting in long-lasting fluorescence and almost unchanged physical form. This demonstrated that phosphor, with excellent characteristics of unsophisticated synthesis, easy preservation and stable fluorescence, showed great potential in light sources, display technology and especially in latent fingerprints visualization on different substrates for forensic science.

## 1. Introduction

Metal nanoclusters exhibit excellent luminescence properties such as molecular electronic transitions, bright luminescence and tunable emission wavelength owing to their ultra-small size and discrete energy levels [1,2,3]. Comparing with noble metal nanoclusters, copper nanoclusters (Cu NCs) are becoming popular research candidates due to the abundant raw materials, low biotoxicity and low cost, which have shown great value in applications of light-emitting devices (LEDs) [4,5,6], chemical sensing [7,8,9], catalysis [10] and biological fields [11,12,13]. However, the vulnerable oxidation by oxygen in the atmosphere and the water absorbing property of Cu NCs powder caused luminescence quenching, greatly limiting their practical application [14].

Some researchers have proposed several methods to improve the stability of the Cu NCs solution. There are mainly three strategies: inducing thiol-protected Cu NCs to aggregate [15,16,17,18], selecting suitable capping ligands [19,20,21,22] and combining Cu NCs with other materials [11,14,23,24,25]. The first strategy employs the aggregation-induced emission (AIE) effect, which is the simplest method compared with the others and it usually requires adding inducers or organic solvent to the Cu NCs solution after synthesis. Such postprocessing would not be affected by the synthesis condition and process of Cu NCs. The current study improves solution stability and the stablized Cu NCs are mainly applied in bioimaging, biosensing, ion detection and material determination. However, research on the stability of Cu NCs powder and their application as phosphor is absent, particularly in latent fingerprints visualization, which is one of the most technologically and extensively used method of identification applications [26,27].

Here, we synthesized Cu NCs powder by two methods and investigated their stability. The stable Cu NCs powder by cation crosslinking method using Ce^3+^ as the crosslinking inducer was first obtained, which was about 100-times more stable than Cu NCs powder synthesized by solvent precipitation method. The time of Ce-Cu NCs powder taken for a 15% drop in luminescence (T_85_) was improved from 2 h to 216 h when the ambient humidity and temperature were 75% and 25 °C, respectively. By several characterizations, binding between Ce^3+^ and carboxyl groups of GSH was verified, which induced the connection of nearby Cu NCs particles. Such compact structure maintained by crosslinking prevented the powder from breaking down by ambient moisture, and Cu NCs along with Ce^3+^ on the edge protected inner Cu NCs from oxygen. The stabilized phosphor was used as latent fingerprints marker, and the fine details of fingerprints on different substrates could be clearly visualized.

## 2. Materials and Methods

### 2.1. Materials and Reagents

Copper(II) nitrate trihydrate (Cu(NO_3_)_2_·3H_2_O), sodium hydroxide (NaOH) and isopropanol alcohol (IPA) were purchased from Hushi (Shanghai, China). Cerium(III) nitrate hexahydrate (Ce(NO_3_)_3_·6H_2_O) and glutathione (GSH) were purchased from Aladdin (Shanghai, China). All chemicals were used without further purification.

### 2.2. Synthesis of Copper Nanoclusters (Cu NCs)

The glutathione-stabilized copper nanoclusters (the GSH-Cu NCs) were synthesized via one-pot ultrasonic method using GSH as the reduction reagent and protective ligands. Briefly, Cu(NO_3_)_2_·6H_2_O solution (1.0 M, 1.2 mL) was added dropwise to GSH solution (0.2 M, 30.0 mL) under vigorous stirring at room temperature for 10 min. Then, NaOH solution (2.0 M) was added to the mixed solution until the pH value was adjusted to 5.0. The obtained solution was treated by ultrasonic waves at a temperature of 25 °C for 20 min. Finally, luminescent Cu NCs were purified with IPA by three centrifugal operations and dissolved in water. The as-prepared Cu NCs were stored at 4 °C for further use.

### 2.3. Synthesis and Stability Investigation of Cu NCs Powder by Solvent Precipitation Method and Cation Crosslinking Method

Cu NCs aqueous solution (0.16 M, 0.25 mL) was added to the mixture of water and IPA (the concentration of IPA was from 0 to 95%). Different quantities of Ce^3+^ in the range of 0.0–4.0 μmol were introduced into Cu NCs solution (0.002 M, 10.0 mL). Fluorescence intensity was monitored by fluorescence spectroscopy at the excitation wavelength of 365 nm, and the absorption spectra were recorded. IPA-Cu NCs powder was obtained from the mixture of Cu NCs and IPA by centrifugation and drying in vacuum oven, and Ce-Cu NCs powder was obtained from the mixture of Cu NCs and Ce^3+^ by centrifugation and drying in vacuum oven. In order to investigate stability, these two phosphors were stored under the same environmental conditions (the ambient humidity of 75% and the temperature of 25 °C), and their PL intensities under the excitation of 365 nm were continuously measured over time. IPA-Cu NCs powder-1 was obtained by drying IPA-Cu NCs powder, which had been stored in the environment for 3 days.

### 2.4. Latent Fingerprints Visualization Based on Ce-Cu NCs Powder

The hands of the donor were washed by water and dried by a hair dryer in advance. The fingers were then pressed on glass, paper, foil and plastic. Ce-Cu NCs powder was carefully sprinkled on four substrates until it covered the fingerprints. Blowball was used to gently remove the extra phosphor. Then, the four fingerprints detection samples were investigated under ultraviolet light of 365 nm.

### 2.5. Characterizations

The transmission electron microscopic (TEM) images were obtained on Tecnai G2 F20 S-Twin (FEI, Portland, OR, USA). UV-vis spectra were recorded on 759S (Shanghai Lengguang, China). Fluorescence spectra were recorded on F97XP (Shanghai Lengguang, China) at the scanning rate of 6000 nm min^−1^. Zeta potential analysis was performed on Malvern Zetasizer Nano ZS90 (Thermo Fisher Scientific, Portland, OR, USA). The synthesized IPA-Cu NCs powder and Ce-Cu NCs powder were characterized by Fourier transform-infrared spectroscopy (FTIR) on Nicolet iS5 (Thermo Scientific, Portland, OR, USA) and X-ray photoelectron spectroscopy (XPS) on K-Alpha (Thermo Scientific, Portland, OR, USA).

## 3. Results and Discussion

**Figure 1:**
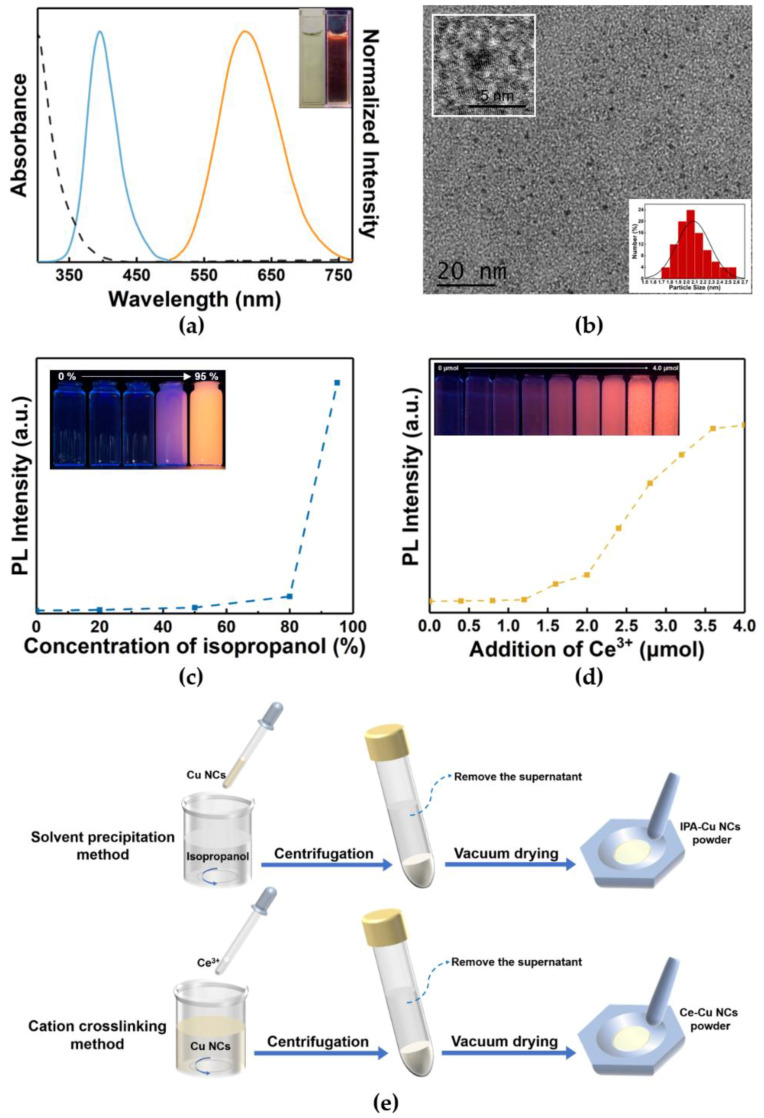
(**a**) Absorption spectrum (dotted line) and PLE spectrum (blue line) at the detection wavelength of 605 nm, PL spectrum (orange line) at the excitation wavelength of 365 nm and (**b**) TEM image of the GSH-Cu NCs; PL intensity at the detection wavelength of 605 nm of Cu NCs aqueous solution (**c**) with different concentrations of IPA (0–95%) and (**d**) with different amounts of Ce^3+^ (0.0–4.0 μmol); (**e**) experimental diagrams of solvent precipitation method and cation crosslinking method. Inset in (**a**) showed the pictures of Cu NCs solution under daylight (the left one) and under UV light (the right one). Inset in (**b**) on the left showed the HR-TEM of Cu NCs and inset on the right showed the particle size distribution of Cu NCs. Insets in (**c**,**d**) showed the photographs of Cu NCs solution under UV light with different concentration of IPA and different quantities of Ce^3+^, respectively.

In this study, water-soluble copper nanoclusters (Cu NCs) were prepared via the one-pot ultrasonic method under room temperature using glutathione (GSH) as the reduction agent and capping ligand. Figure 1a showed the absorption, excitation, and emission spectrum of the as-synthesized Cu NCs. No peak at 507 nm was found in the absorption spectrum, indicating that large copper nanoparticles were not generated [28]. PL and PLE spectrum peaks were located at 395 nm and 605 nm, respectively. The little overlap between absorption spectrum and PL spectrum of Cu NCs showed a large Stokes shift. The faint yellow transparent Cu NCs aqueous solution emitted weak orange light under the excitation of 365 nm light (Inset in Figure 1a). The dispersive spherical particles with an average size of 2.08 nm were observed in TEM images (Figure 1b). The XPS analysis was conducted to verify the elemental composition of Cu NCs and the valence state of Cu. Element components of Cu, O, N, C and S were confirmed by their binding energy signals (Figure S1a). From the XPS survey spectrum of Cu 2p, two obvious peaks at 932 eV and 952 eV assigned to 2p_1/2_ and 2p_2/3_ electrons of Cu were observed (Figure S1b). The absence of the signal peak at 942 eV proved that Cu^2+^ was reduced by GSH. Significantly, the difference between the typical 2p_3/2_ peaks of Cu(0) and Cu(I) was only 0.1 eV, indicating that the valence state of Cu in the obtained Cu NCs was likely to be between 0 (in the core) and +1 (at the surface) [29,30,31]. FTIR spectra were employed to figure out the surface properties of the as-prepared Cu NCs (Figure S2). The peak in the FTIR spectrum of GSH at 2524 cm^−1^ corresponded to the S-H stretching vibration disappeared in the spectrum of Cu NCs, indicating that GSH was coated on the surface of Cu NCs through Cu-S bonding [32]. These characterizations proved that the GSH-Cu NCs were successfully prepared.

An organic solvent isopropanol (IPA) and a rare earth cation Ce^3+^ were introduced into GSH-Cu NCs. As demonstrated in Figure 1c,d, the fluorescence intensity of Cu NCs was on increasing trends when Cu NCs were dissolved in different concentrations of IPA or introduced different amounts of Ce^3+^. Meanwhile, transparent Cu NCs solutions gradually became turbid where white suspension emerged and the faint fluorescence turned into intense orange emission from the insets of Figure 1c,d. From the PL spectra of Cu NCs solutions in Figures S3 and S4, obvious blue shifts of the emission peaks were observed with the addition of IPA and Ce^3+^ due to the increasing average Cu(I) ••• Cu(I) distance caused by the intermolecular coprophilic attraction between the nearby Cu NCs [33]. Furthermore, the absorption peaks also became broader with the increase in IPA and Ce^3+^, revealing the formation and growth of aggregates (Figures S5 and S6) [34]. The aggregation structure on one hand facilitated the ligand-to-metal charge transfer (LMCT) and/or ligand-to-metal-metal charge transfer (LMMCT), resulting in a metal-centered triplet state radiative transition [5] and, on the other hand, suppressed molecule rotation and vibration, resulting in the fluorescence enhancement of Cu NCs solution [35]. The aggregation-induced emission effect (the AIE effect) phenomenon of Cu NCs in solution induced by the solvent IPA and rare earth cation Ce^3+^ were consistent with previous studies [16,36].

**Figure 2:**
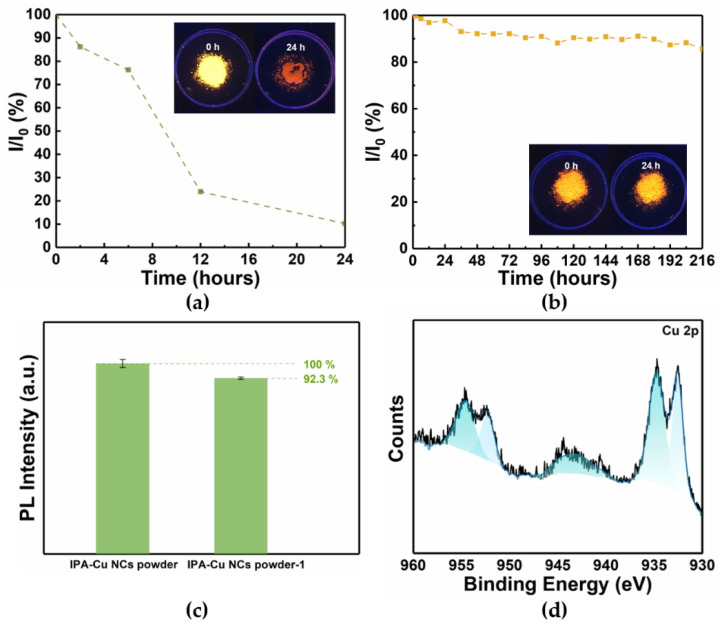
(**a**) PL intensity ratios (I/I_0_, I and I_0_ represented PL intensity after different hours and initial PL intensity, respectively) of IPA-Cu NCs powder at the detection wavelength of 605 nm (365 nm excitation); (**b**) PL intensity ratios (I/I_0_, I and I_0_ represented PL intensity after different hours and initial PL intensity respectively) of Ce-Cu NCs powder at the detection wavelength of 605 nm (365 nm excitation); (**c**) initial PL intensity of IPA-Cu NCs powder and PL intensity of IPA-Cu NCs powder-1 at the detection wavelength of 605 nm; (**d**) XPS spectrum of Cu 2p electrons of IPA-Cu NCs powder-1. Insets in (**a**,**b**) show photographs under UV light of IPA-Cu NCs powder and Ce-Cu NCs powder stored under the ambient humidity of 75% and temperature of 25 °C after 24 h.

We introduced solvent precipitation method and cation crosslinking method to synthesize Cu NCs powder. As Figure 1e showed, the Cu NCs solution was added to a large amount of IPA in order to obtain precipitation, and they were centrifuged and dried under vacuum to obtain IPA-Cu NCs powder. Ce-Cu NCs powder was obtained from the suspended precipitate of Cu NCs solution with Ce^3+^ by centrifugation and vacuum drying. In order to investigate solid stability, the fluorescence intensities of these two phosphors were recorded after incubation for different times under the same condition. The fluorescence intensity of IPA-Cu NCs powder dropped rapidly and fell to 10% of the initial intensity only 24 h later (Figure 2a). At the same time, the powder partly melted after 6 h and turned into a gelatinous paste 24 h later (Figure S7). While the fluorescence intensity of Ce-Cu NCs powder remained more than 85% of its initial intensity after 216 h and the morphology hardly changed. The time taken for a 15% drop in luminescence (T_85_) was improved from 2 h to 216 h, showing prominent stability compared with IPA-Cu NCs powder (Figure 2b). In order to verify the prime factor that caused the fluorescence decline, IPA-Cu NCs powder exposed in the environment for 3 days was redried to determine PL intensity, and IPA-Cu NCs powder-1 regained 92.3% of its initial fluorescence intensity (Figure 2c). The well-recovered fluorescence of the aged Cu NCs powder by redrying implied the strong impact of ambient moisture on the stability of IPA-Cu NCs powder. Pictures of IPA-Cu NCs powder under different humidity after different times supported the inference are provided from which more severe water absorption and luminescence quenching of the powder under higher humidity could be observed in Figure S7. Meanwhile, Ce-Cu NCs powder exhibited greater stability under different humidity (Figure S7). IPA-Cu NCs powder was obtained due to the steeply decreasing dissolution of GSH-Cu NCs during organic solvent-evaporating processes; thus, aggregation structure easily collapsed when the water-soluble Cu NCs powder absorbed water from an environment with high humidity [16]. In the XPS spectrum of Cu 2p electrons in IPA-Cu NCs powder-1, the signal peaks at 934.7 eV, 943.3 eV and 954.5 eV represented the signal of the Cu(II) characteristic peak, which showed that oxidation also affects the fluorescence stability in addition to water absorption (Figure 2d) [37,38,39,40].

**Figure 3:**
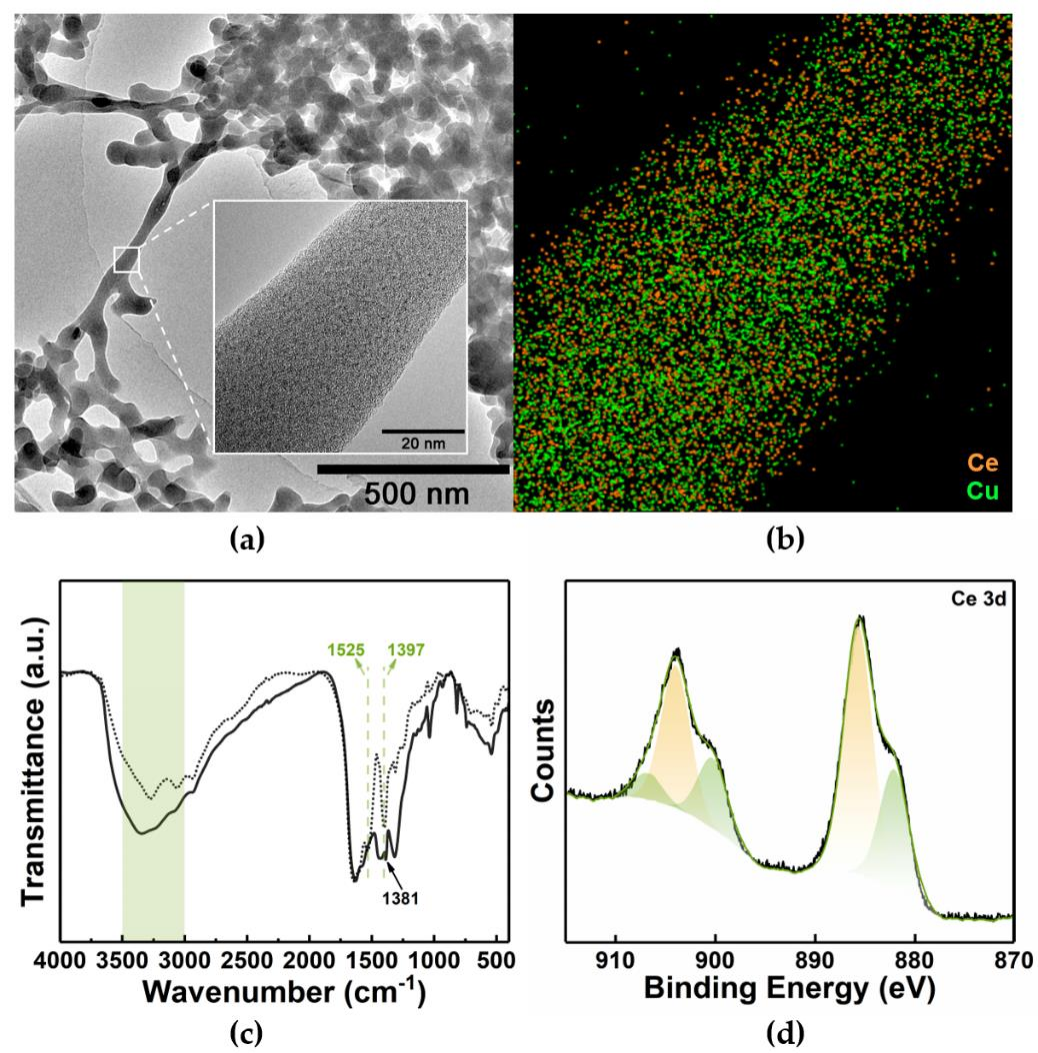
(**a**) TEM image of Ce-Cu NCs aggregates; (**b**) EDS elemental mapping of Cu and Ce in Ce-Cu NCs aggregates; (**c**) FTIR spectra of Cu NCs (dotted line) and Ce-Cu NCs aggregates (solid line); (**d**) XPS spectrum of Ce 3d electrons in Ce-Cu NCs aggregates. Inset in (**b**) shows zoomed-in detail of Ce-Cu NCs aggregates TEM image.

Several characterizations were carried out to figure out the interaction mechanism of cerium (III) and Cu NCs. The microscopic morphology of the mixture of Cu NCs and Ce^3+^ (Ce-Cu NCs aggregates) was investigated by TEM. There was an intricate network structure generated after adding Ce^3+^ into Cu NCs (Figure 3a), which was completely different from the well-dispersed sphere structure of bare Cu NCs shown in Figure 1b. The EDS elemental mappings and HADDF TEM were performed to investigate the combination between Ce^3+^ and Cu NCs (Figures S8 and S9a). Cu, Ce, and other expected elements observed in EDS mapping images (Figure 3b and Figure S9b–f) were homogeneously distributed in Ce-Cu NCs aggregates, indicating that Ce^3+^ and Cu NCs were fully crosslinked together.

From the FTIR spectra of Cu NCs and Ce-Cu NCs aggregates, the broad peaks around 3000–3500 cm^−1^ represented -NH- and -OH stretching vibration. -COOH stretching vibration at 1525 cm^−1^ in Cu NCs was absent after adding Ce^3+^ and a signal at 1381 cm^−1^ corresponding to Ce-O bond appeared in Ce-Cu NCs aggregates (Figure 3c), which indicated that Ce^3+^ were likely to anchor themselves to -COOH on the capping ligands of Cu NCs through Ce-O bonding [41,42]. Moreover, the stretching vibration of -COOH at 1397 cm^−1^ in Cu NCs shifted to a higher wavenumber in Ce-Cu NCs aggregates [42]. Since Ce^3+^ had a low standard electrode potential of −2.32, it could easily become oxidized and donated electron pairs to form coordinate bonds [43]. Therefore, -COOH groups could have accepted the external charge, and vibration frequency also increased, resulting in a shift of the absorption band toward a higher wavenumber. The Zeta potential characterization was employed to measure the surface charge of Cu NCs and Ce-Cu NCs aggregates (Figure S10). The surface potential of the bare Cu NCs turned from negative to positive after adding Ce^3+^. This reversion was caused by the addition of Ce^3+^, which coordinated with the carboxyl groups of the surface molecules via electrostatic interaction.

The XPS survey of Ce-Cu NCs aggregates is shown in Figure S11a. According to the XPS survey of Cu in the aggregates, the valence state of Cu remained the same as that of Cu in Cu NCs, indicating that Ce^3+^ did not interact with the metal cores (Figure S11b). Moreover, there were no Cu(II) signal peaks in the Cu 2p XPS spectrum of Ce-Cu NCs aggregates stored for 3 days, revealing that the powder was much more stable to oxygen than IPA-Cu NCs powder (Figure S12). The Ce 3d XPS survey spectrum could be divided into five peaks at 885.66 eV, 903.95 eV, 882.04 eV, 900.25 eV and 906.73 eV (Figure 3d). The peaks at 885.66 eV and 903.95 eV represented Ce^3+^ 3d_3/2_, and the other three peaks stood for Ce^4+^ 3d_5/2_ [41]. The result that the valence state of Ce^3+^ raised from +3 to +4 partially in Ce-Cu NCs aggregates was consistent with the shift of -COOH stretch mode from the FTIR spectra of Cu NCs and Ce-Cu NCs aggregates. We infer that Ce^3+^ and the carboxyl groups of the capping ligands could form coordinate bonds, and part of Ce^3+^ tended to be oxidized via donating electron pairs to ligand molecules.

**Scheme 1:**
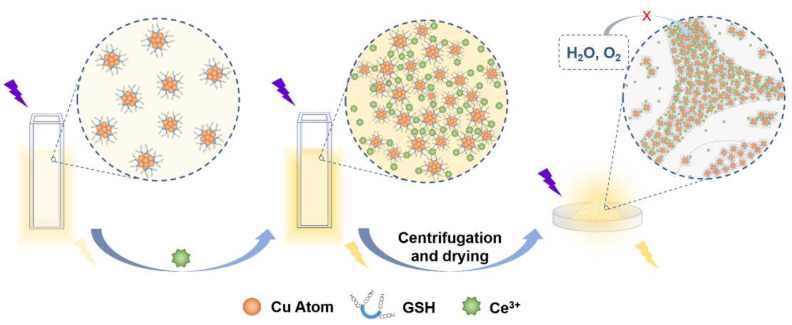
Schematic illustration of the interaction between Ce^3+^ and Cu NCs in Ce-Cu NCs powder and latent fingerprints visualization using phosphor powder.

According to the results mentioned above, the interaction between Ce^3^+ and Cu NCs in the stable Ce-Cu NCs powder was depicted in Scheme 1. Cu NCs were monodisperse in clarified aqueous solution, which emitted faint orange light excited by 365 nm light. Electrostatic repulsion caused by carboxyl groups of capping ligands prevented Cu NCs from aggregating [11]. While after adding a specific amount of Ce^3+^, it worked as the crosslinking inducer in the process that preferred combining with carboxyl groups on Cu NCs. Bonds between Ce^3+^ and ligand molecules induced nearby Cu NCs to connect and aggregate, which induced the aggregates to precipitate. Furthermore, such tight structure maintained by crosslinking prevented the aggregates from absorbing ambient moisture, and Cu NCs along with Ce^3+^ on the edge of the aggregates protected the inner Cu NCs from oxygen. Hence, Ce-Cu NCs powder exhibited greater stability in both physical form and luminescence intensity.

**Figure 4:**
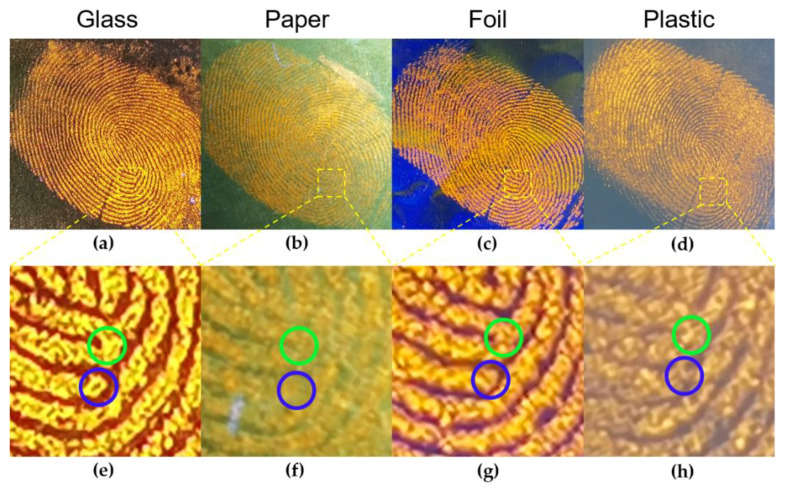
(**a**–**d**) Photos of latent fingerprints visualization using Ce-Cu NCs powder under UV light. (**e**–**h**) The zoomed-in details of labeled fingerprints in which the ridge termination points (green circle) and the bifurcation points (blue circle) were marked. Fingerprints were pressed on glass (**a**,**e**), paper (**b**,**f**), foil (**c**,**g**) and plastic (**d**,**h**).

Compared with Cu NCs powder obtained by solvent precipitation method, Cu NCs powder by cation crosslinking method was easier to grind into fine powder and to preserve for a long time, which enhanced its practical use value as a phosphor. Moreover, the synthesis of Cu NCs powder by cation crosslinking would not waste a large amount of antisolvent, which was more suitable and more convenient for mass preparation than solvent precipitation method. Owing to photoluminescence, low-toxicity, stable and low-cost properties, Ce-Cu NCs powder showed great potential in latent fingerprints visualization in which Cu NCs were rarely used. Latent fingerprints referring to the prints left on items by touching of human hands can hardly be seen with naked eyes [44,45,46,47]. The visualization of latent fingerprints is significant in forensic investigation due to the uniqueness of fingerprints for individual identification [48,49]. Latent fingerprints on glass, paper, foil and plastic marked by Ce-Cu NCs powder were investigated under UV light (Figure 4a–d). The legible orange-emitting fingerprints images under UV light demonstrated that Ce-Cu NCs powder could favorably adhere to the oily sebum of human fingers. Latent fingerprints on the substrates without phosphor treating (Figure S13) were hard to recognize with the naked eye, but the ridge termination points, bifurcation points and scar were clearly visible after the latent fingerprints were labelled by Ce-Cu NCs powder (Figure 4e–h and Figure S14). Ce-Cu NCs powder showed high fineness and great ability to enhance the contrast of the fingerprints pattern and different substrates background. Furthermore, the visualized latent fingerprints on different substrates made it possible to apply phosphor at real crime scenes with the integrated automated fingerprint identification system (IAFIS) test [50]. The use of Ce-Cu NCs powder on detailed latent fingerprints visualization broadened the path for the application of Cu NCs and provided strong support for forensic investigation.

## 4. Conclusions

We introduced a novel cation crosslinking method to obtain stable Cu NCs powder. A rare earth metal cation Ce^3+^ was used as an inducer linked to the carboxyl groups on surface ligand GSH through electrostatic interaction. Such tight structure restricted the rotation and vibration of GSH molecules and helped protect Ce-Cu NCs powder against ambient moisture, contributing to a 100-fold increase in solid stability compared with the IPA-Cu NCs powder obtained via solvent precipitation method. Fine latent fingerprints visualization on different substrates was achieved by applying the stabled phosphor showing great potential in forensic investigation.

## Data Availability

The data presented in this study are available upon request from the corresponding author.

## Supplementary Materials

The following are available online at https://www.mdpi.com/article/10.3390/nano11123371/s1, Figure S1. (a) XPS survey of GSH-Cu NCs; (b) XPS spectrum of Cu 2p electrons in GSH-Cu NCs; Figure S2. FTIR spectrum of GSH and Cu NCs; Figure S3. Emission spectrum of Cu NCs with different concentration of IPA (from 0 to 95%) under the excitation of 365 nm; Figure S4. Emission spectrum of Cu NCs with different amount of Ce^3+^ (from 0 to 4.0 μmol) under the excitation of 365 nm; Figure S5. Absorption spectrum of Cu NCs with different concentration of IPA (from 0 to 95%); Figure S6. Absorption spectrum of Cu NCs with different amount of Ce^3+^ (from 0 to 4.0 μmol); Figure S7. Photographs of IPA-Cu NCs powder and Ce-Cu NCs powder after different time (0, 6 and 24 h) in the same store condition (temperature of 25 ℃) under daylight and under UV light under different ambient humidity: (a) 11%, (b) 30%, (c) 57% and (d) 75%; Figure S8. Map sum spectrum of Ce-Cu NCs aggregates; Figure S9. (a) HAADF-STEM image of Ce-Cu NCs aggregates; EDS elemental mapping of (b) C, (c) N, (d) O, (e) S and (f) overlapped EDS images of Ce-Cu NCs aggregates; Figure S10. Zeta potential of Cu NCs and Ce-Cu NCs aggregates; Figure S11. (a) XPS survey of Ce-Cu NCs aggregates; (b) XPS spectrum of Cu 2p electrons in Ce-Cu NCs aggregates; Figure S12. XPS spectrum of Cu 2p electrons in Ce-Cu NCs aggregates stored for 3 days; Figure S13. Photos under daylight of latent fingerprints on (a) glass, (b) paper, (c) foil, and (d) plastic; Figure S14. (a)Photo of finger of the donor under daylight; (b) photo of latent fingerprints detection using Ce-Cu NCs powder under UV light. Inset in (b) showed the scar of the donor’s finger.
