# Supplementary material for: Cation Crosslinking-Induced Stable Copper Nanoclusters Powder as Latent Fingerprints Marker
Source: Nanomaterials (Basel). 2021 Dec 12;11(12):3371. doi: 10.3390/nano11123371 (PMC8708820; doi:10.3390/nano11123371)
Supplement: Supplementary file 1 [file nanomaterials-11-03371-s001.zip › nanomaterials-1455996-SI.pdf]

nanomaterials

Supplementary Materials

Cation Crosslinking-Induced Stable Copper Nanoclusters Powder as Latent Fingerprints Marker

Yi Qiu [1], Zhuoqi Wen [2], Shiliang Mei [1], Jinxin Wei [1], Yuanyuan Chen [1], Zhe Hu [1], Zhongjie Cui [1], Wanlu Zhang [1], Fengxian Xie [1] and Ruiqian Guo [1,2,3,4,*]

1 Institute for Electric Light Sources, School of Information Science and Technology, Fudan University, Shanghai 200433, China; 19210720021@fudan.edu.cn (Y.Q.); meishiliang@fudan.edu.cn (S.M.); 20210720024@fudan.edu.cn (J.W.); 19210720022@fudan.edu.cn (Y.C.); 20110720020@fudan.edu.cn (Z.H.); 19110720020@fudan.edu.cn (Z.C.); fdwlzhang@fudan.edu.cn (W.Z.); xiefengxian@fudan.edu.cn (F.X.)
2 Institute of Future Lighting, Academy for Engineering and Technology, Fudan University, Shanghai 200433, China; 19110860003@fudan.edu.cn
3 Zhongshan-Fudan Joint Innovation Center, Zhongshan 528437, China
4 Yiwu Research Institute of Fudan University, Chengbei Road, Yiwu City, Zhejiang, 322000, China
* Correspondence: rqguo@fudan.edu.cn

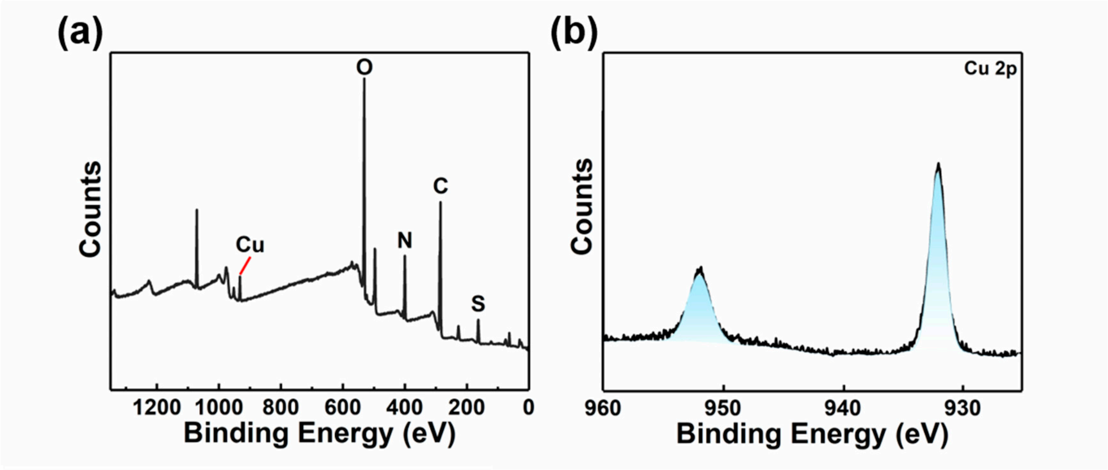

Figure S1. (**a**) XPS survey of GSH-Cu NCs; (**b**) XPS spectrum of Cu 2p electrons in GSH-Cu NCs.

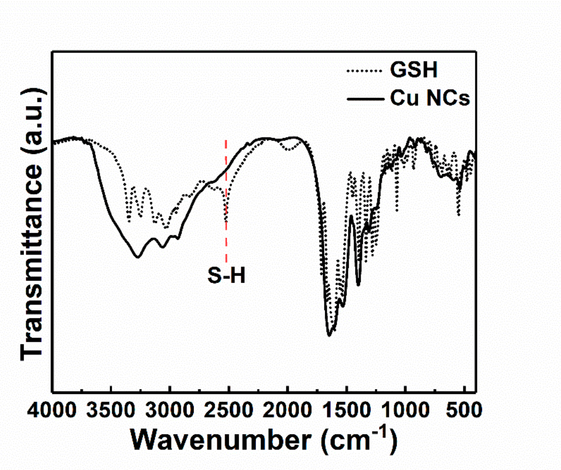

Figure S2. FTIR spectrum of GSH and Cu NCs.

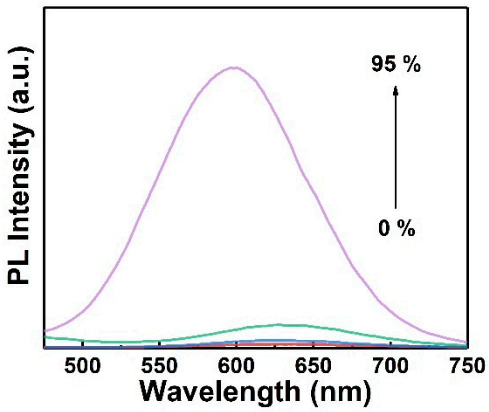

Figure S3. Emission spectrum of Cu NCs with different concentration of IPA (from 0 to 95 %) under the excitation of 365 nm.

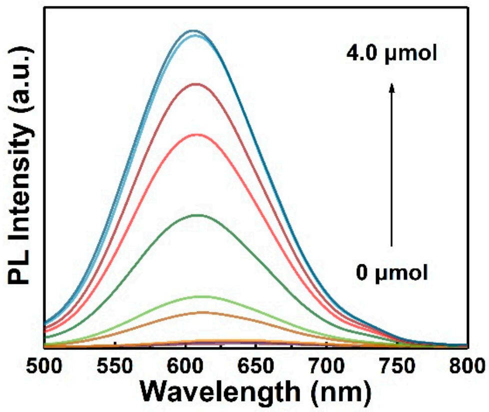

Figure S4. Emission spectrum of Cu NCs with different amount of Ce^{3+} (from 0 to 4.0 μmol) under the excitation of 365 nm.

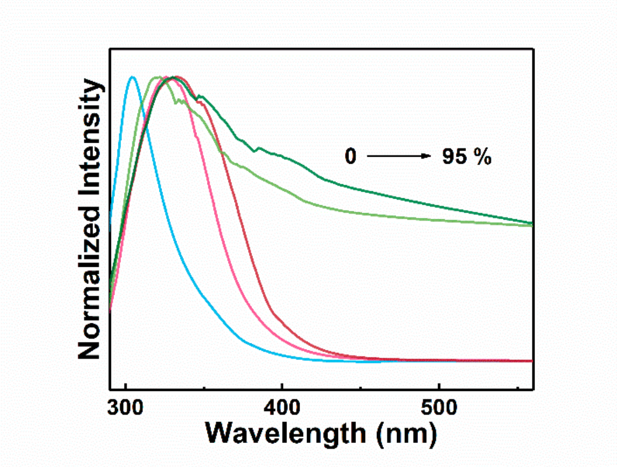

Figure S5. Absorption spectrum of Cu NCs with different concentration of IPA (from 0 to 95 %).

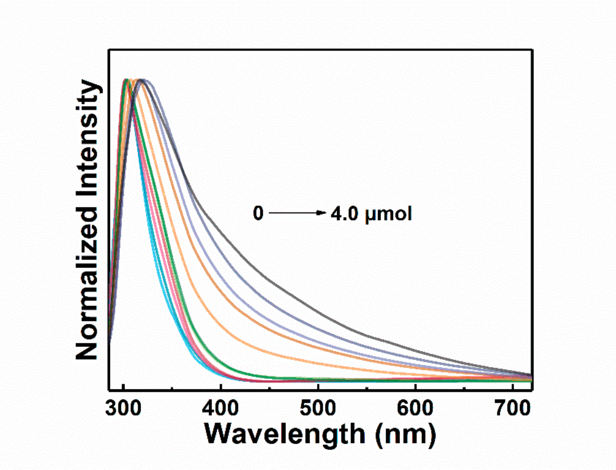

Figure S6. Absorption spectrum of Cu NCs with different amount of Ce^{3+} (from 0 to 4.0 μmol).

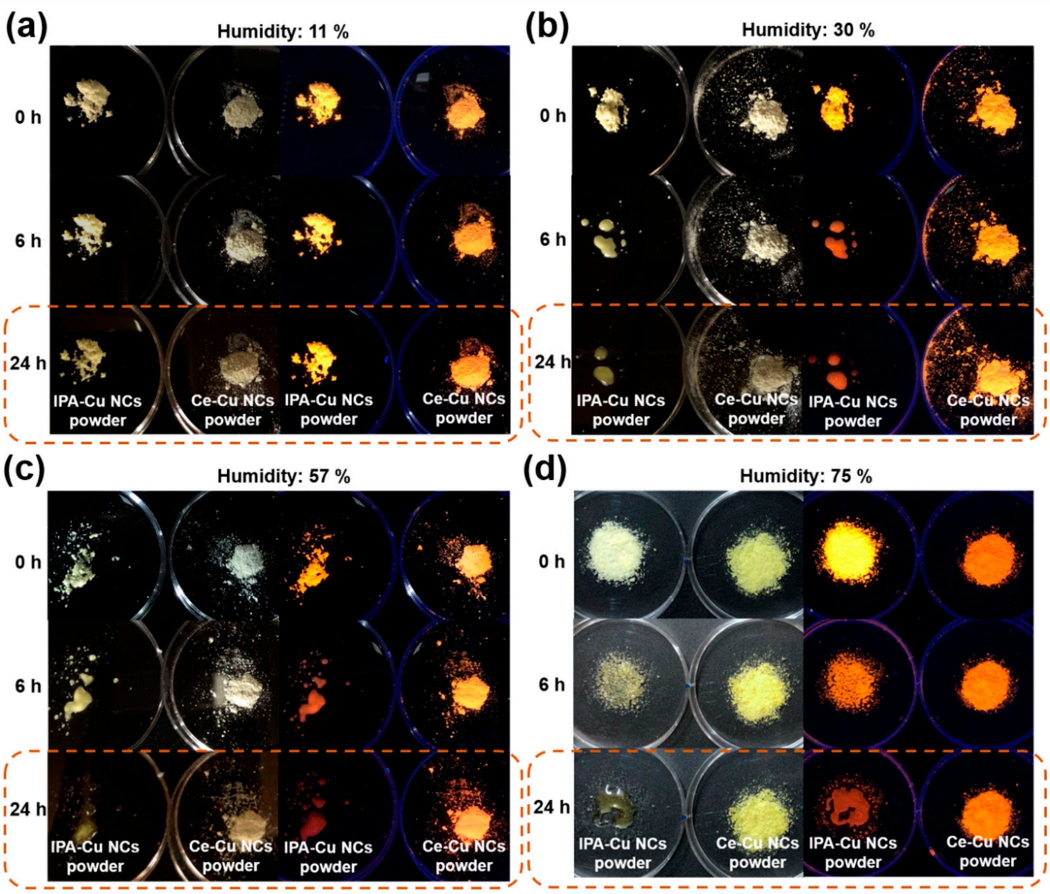

Figure S7. Photographs of IPA-Cu NCs powder and Ce-Cu NCs powder after different time (0, 6 and 24 h) in the same store condition (temperature of 25 °C) under daylight and under UV light under different ambient humidity: (**a**) 11 %, (**b**) 30 %, (**c**) 57 % and (**d**) 75 %.

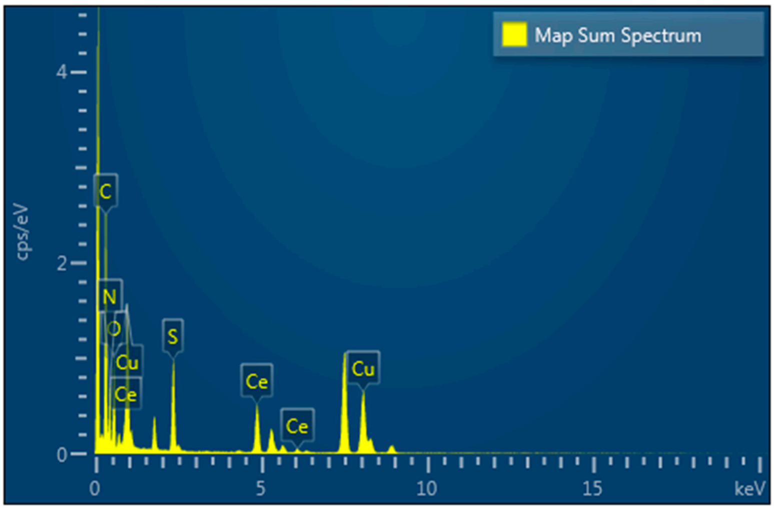

Figure S8. Map sum spectrum of Ce-Cu NCs aggregates.

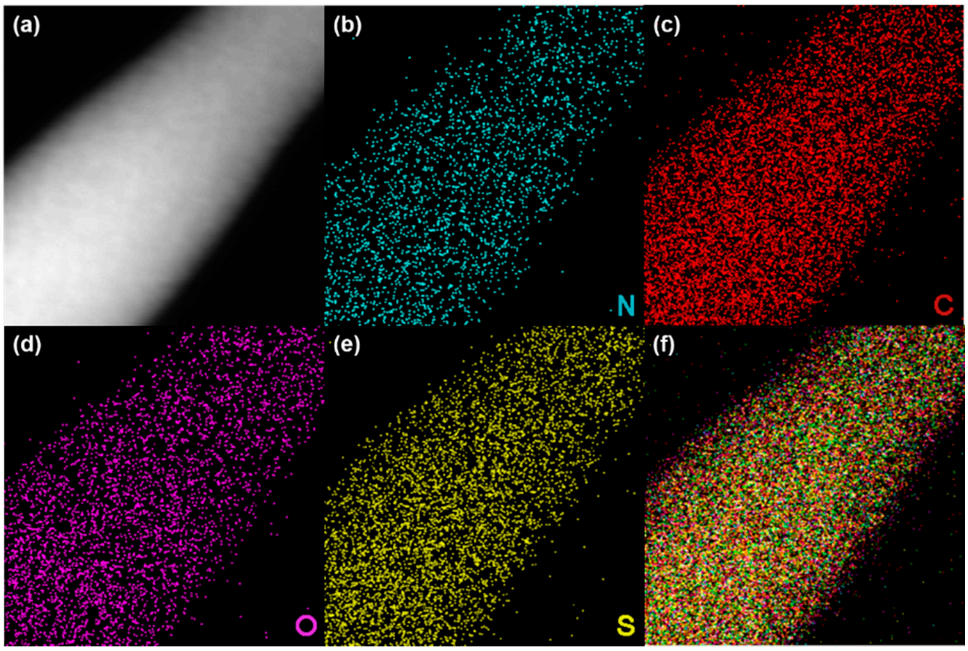

Figure S9. (**a**) HAADF-STEM image of Ce-Cu NCs aggregates; EDS elemental mapping of (**b**) C, (**c**) N, (**d**) O, (**e**) S and (**f**) overlapped EDS images of Ce-Cu NCs aggregates.

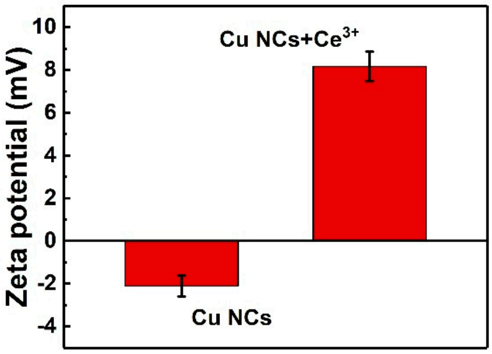

Figure S10. Zeta potential of Cu NCs and Ce-Cu NCs aggregates.

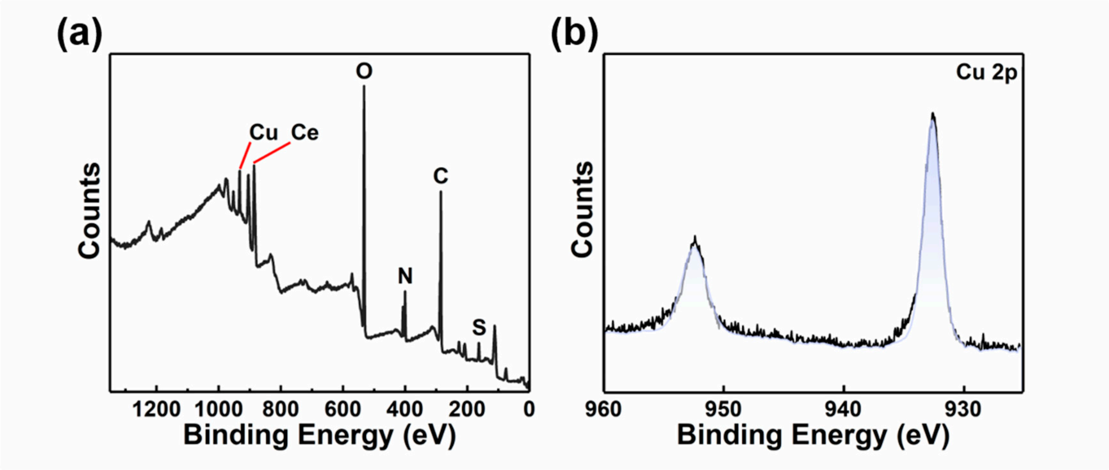

Figure S11. (**a**) XPS survey of Ce-Cu NCs aggregates; (**b**) XPS spectrum of Cu 2p electrons in Ce-Cu NCs aggregates.

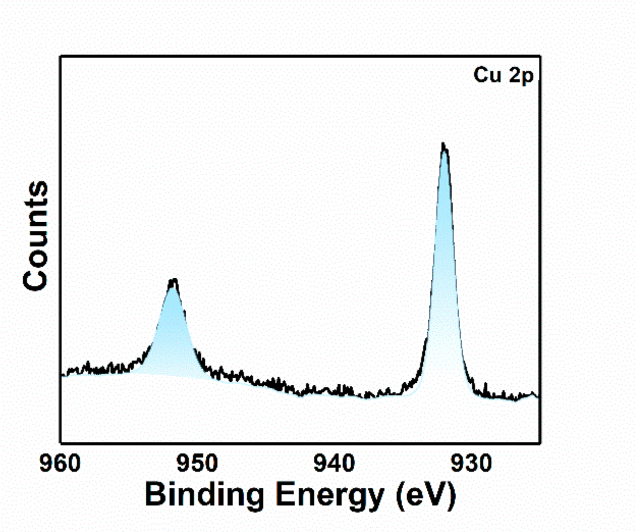

Figure S12. XPS spectrum of Cu 2p electrons in Ce-Cu NCs aggregates stored for 3 days.

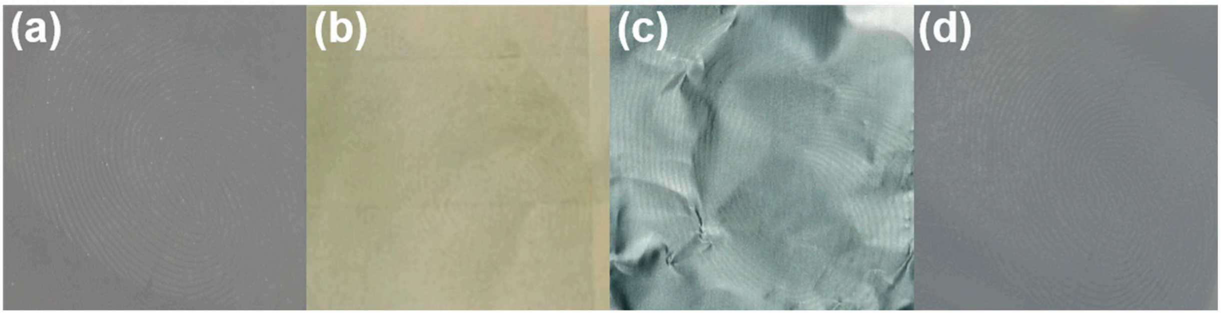

Figure S13. Photos under daylight of latent fingerprints on (**a**) glass, (**b**) paper, (**c**) foil, and (**d**) plastic.

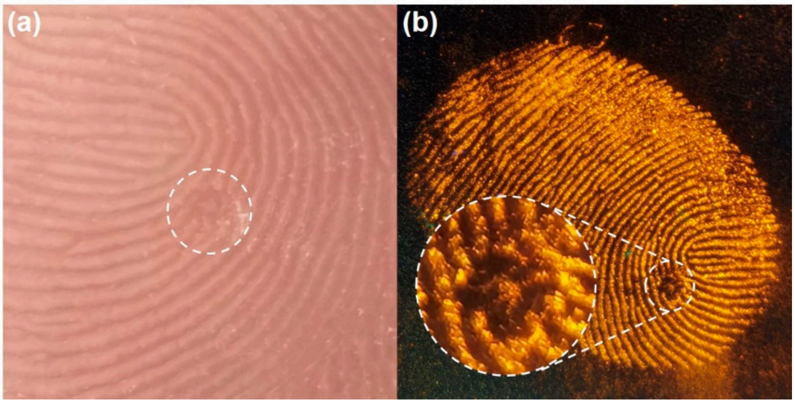

Figure S14. (**a**)Photo of finger of the donor under daylight; (b) photo of latent fingerprints detection using Ce-Cu NCs powder under UV light. Inset in (**b**) showed the scar of the donor's finger.
